# Hysteretic hERG channel gating current recorded at physiological temperature

**DOI:** 10.1038/s41598-022-10003-7

**Published:** 2022-04-08

**Authors:** David K. Jones

**Affiliations:** 1 Department of Pharmacology, University of Michigan School of Medicine, Ann Arbor, MI 48109 USA; 2 Department of Internal Medicine, University of Michigan School of Medicine, Ann Arbor, MI 48109 USA

**Keywords:** Ion transport, Patch clamp

## Abstract

Cardiac hERG channels comprise at least two subunits, hERG 1a and hERG 1b, and drive cardiac action potential repolarization. hERG 1a subunits contain a cytoplasmic PAS domain that is absent in hERG 1b. The hERG 1a PAS domain regulates voltage sensor domain (VSD) movement, but hERG VSD behavior and its regulation by the hERG 1a PAS domain have not been studied at physiological temperatures. We recorded gating charge from homomeric hERG 1a and heteromeric hERG 1a/1b channels at near physiological temperatures (36 ± 1 °C) using pulse durations comparable in length to the human ventricular action potential. The voltage dependence of deactivation was hyperpolarized relative to activation, reflecting VSD relaxation at positive potentials. These data suggest that relaxation (hysteresis) works to delay pore closure during repolarization. Interestingly, hERG 1a VSD deactivation displayed a double Boltzmann distribution, but hERG 1a/1b deactivation displayed a single Boltzmann. Disabling the hERG 1a PAS domain using a PAS-targeting antibody similarly transformed hERG 1a deactivation from a double to a single Boltzmann, highlighting the contribution of the PAS in regulating VSD movement. These data represent, to our knowledge, the first recordings of hERG gating charge at physiological temperature and demonstrate that VSD relaxation (hysteresis) is present in hERG channels at physiological temperature.

## Introduction

The *KCNH2* gene encodes the hERG 1 voltage-gated potassium channel that conducts the critical repolarizing current I_Kr_^1,2^. *KCNH2* mutations that reduce I_Kr_ or off-target hERG 1 channel block cause the cardiac disorder long QT syndrome (LQTS) and increase the likelihood for cardiac arrhythmia and sudden cardiac death^2,3^.

hERG channels are tetrameric with a single, central pore that is gated by the movements of the voltage sensing domain (VSD) of each subunit^4,5^. It is the movement of the VSDs in response to changes in the membrane electric field that triggers pore opening or closure in voltage-dependent channels, including hERG channels^6–11^. Like other voltage-sensitive channels, hERG VSDs transition from their active state to a more energetically favorable “relaxed” state during prolonged depolarization (> 300 ms at room temperature)^9,12–15^. VSD relaxation hyperpolarizes the voltage dependence of VSD deactivation and delays pore closure upon repolarization^11,12,14,16^. Whether VSD relaxation occurs at physiological temperature is unknown, but if present, would presumably work to enhance hERG 1 channel open probability during cardiac repolarization, by delaying pore closure.

Native hERG 1 channels comprise at least two subunits, hERG 1a and hERG 1b^17–21^ that are identical with the exception of their respective N-termini^19,20^. The hERG 1a N-terminal domain contains a Per-Arnt-Sim (PAS) domain^22^, which modulates hERG 1 gating through interactions between the cytoplasmic linker of the 4th and 5th transmembrane helices and the C-terminal cyclic nucleotide binding homology domain (CNBHD)^23–27^. Mutations within the hERG 1a PAS domain that disrupt the PAS/CNBHD interaction accelerate channel activation and deactivation, and disrupt inactivation^23,28–33^. Disrupting the PAS/CNBHD interaction also accelerates VSD deactivation and decreases the hysteresis of ionic current^13,15,16^. Surprisingly, the reports of PAS disruption on VSD hysteresis are mixed, where two reports demonstrated reduced hysteresis^13,15^, but a third reported that VSD hysteresis remained intact^16^. hERG 1b subunits have a unique N-terminal domain that lacks a PAS domain. Co-expression of the PAS-deficient hERG 1b subunit with hERG 1a reduces the relative number of PAS domains per channel, and thus heteromeric hERG 1a/1b channels display accelerated channel kinetics and larger currents compared to hERG 1a homomeric channels^34,35^. Gating charge from heteromeric hERG 1a/1b channels has not been recorded.

Here we report hERG gating charge recorded from hERG 1a and hERG 1a/1b channels at near physiological temperatures (36 ± 1 °C). We found that hERG 1 VSD relaxation is intact in homomeric hERG 1a channels and heteromeric hERG 1a/1b channels at physiological temperatures. Surprisingly, hERG 1a/1b channels displayed enhanced gating charge hysteresis, reflecting a stabilized relaxed state, compared to homomeric hERG 1a channels. PAS disruption, using a PAS targeting antibody on homomeric channels transformed homomeric hERG 1a gating charge and ionic current to a phenotype similar to that observed in heteromeric hERG 1a/1b channels. These data provide a more in-depth mechanism for the differential gating between homomeric hERG 1a channels and heteromeric hERG 1a/1b channels. These data also demonstrate that VSD relaxation is intact in hERG 1 channels at physiological temperature.

## Results

### hERG 1a gating current at physiological temperature

**Figure 1:**
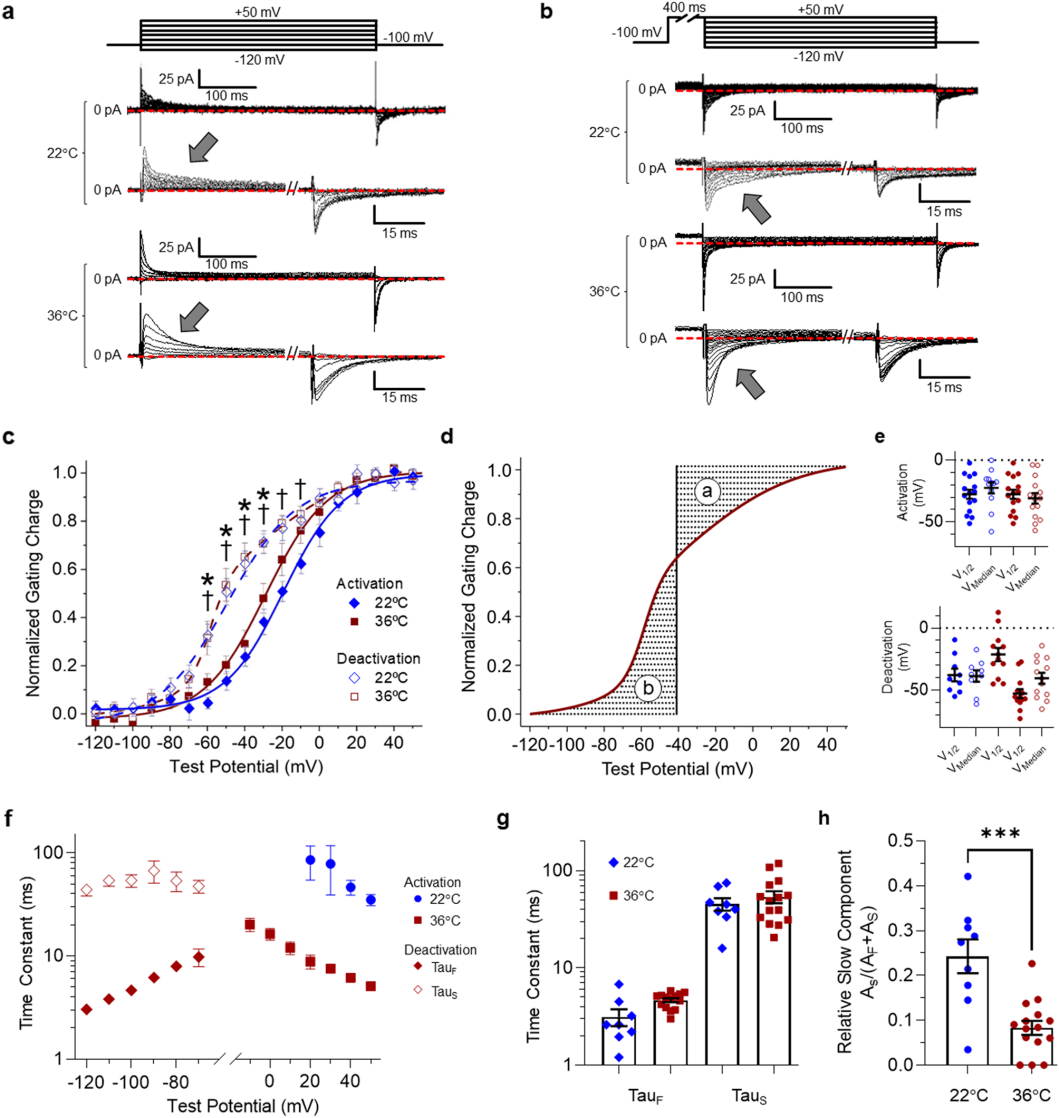
Hysteretic Behavior of hERG 1a gating charge at room temperature (22 °C) and near physiological temperature (36 °C). (**a**) The pulse protocol (top) and corresponding activation gating current traces recorded at 22 °C and 36 °C. An expanded view of the current traces is shown at bottom. The voltage dependence of hERG 1a gating charge activation was measured by recording gating currents elicited by depolarization from a negative (− 100 mV) holding potential. (**b**) The pulse protocol (top) and corresponding deactivation gating current traces (middle). An expanded view of the current traces is shown at bottom. The voltage dependence of hERG 1a gating charge deactivation was measured by recording gating currents elicited by hyperpolarization from a positive (+ 50 mV) holding potential. (**c**) Voltage dependence of hERG 1a voltage sensor activation (filled symbols) and deactivation (open symbols) recorded at 22 °C (blue) and 36 °C (red). Normalized gating charge elicited during the test pulse (arrows) normalized, plotted as a function of test potential, and fitted with either a single Boltzmann (activation) or a double Boltzmann (deactivation) function. The majority of gating charge deactivation occurred at membrane potentials significantly more negative than activation. † indicates *p* < 0.05 between deactivation and activation at 22 °C. * indicates *p* < 0.05 between activation and deactivation at 36 °C. (**d**) Graphical representation of the median voltage of charge movement (V_median_). The shaded areas “*a*” and “*b*” represent the area between the curve and the ordinate axis. The V_median_ is the point along the X axis where the absolute value of *a* and *b* are equal. (**e**) V_1/2_ values and V_median_ values for VSD activation (top) and deactivation (bottom) recorded at 22 °C (blue) and 36 °C (red). Two V_1/2_ values are reported for deactivation at 36 °C, reflecting the two components of the double Boltzmann. (**f**–**h**) Time constants of gating current decay are hysteretic. We fitted the decay of gating currents elicited during each test pulse with a single (activation) or double exponential function (deactivation). (**g**) Slow and fast time constants of gating current decay during a deactivating pulse to − 100 mV. (**h**) Relative magnitude of the slow component of gating current decay at − 100 mV. All data are reported as mean ± SEM. *** indicates *p* < 0.001. *n* = 6–12.

In this study, we sought to characterize hERG 1 hysteresis at near physiological temperatures (36 ± 1 °C). We first measured hERG 1a gating current activation and deactivation from HEK293 cells stably expressing the hERG 1a subunit at both room temperature (22 °C) and 36 °C (Fig. 1). To isolate the hERG 1 gating current from ionic current, we replaced the monovalent cations of the bath and pipette solutions with NMDG^+^ and TEA^+^, respectively^36^. We measured the voltage dependence of VSD activation by recording the gating current elicited during a 400 ms test potential from a − 100 mV holding potential (Fig. 1a). We measured the voltage dependence of VSD deactivation by recording the gating current elicited during a 400 ms hyperpolarizing test pulse from a 400 ms, + 50 mV conditioning pre-pulse to fully activated all of the channels (Fig. 1b). We selected 400 ms for all pre-pulses because of its physiological relevance to the human cardiac action potential. The gating currents recorded during each test pulse were integrated, and the resultant gating charge was plotted as a function of test potential and fitted with a Boltzmann function (Fig. 1c). Voltage sensor activation and deactivation at 22 °C displayed a single Boltzmann distribution (Eq. 1, Fig. 1c,e, Supplementary Table S1). Similarly, voltage sensor activation at 36 °C displayed a single Boltzmann distribution (Eq. 1, Fig. 1c,e, Supplementary Table S1). Surprisingly, voltage sensor deactivation at 36 °C displayed a double Boltzmann distribution (Eq. 2). Roughly 30% of the gating charge deactivated with a V_1/2_ nearly identical to that measured for VSD activation (− 21.3 ± 5.3 mV), and the remaining 70% deactivated with a hyperpolarized V_1/2_ (− 52.8 ± 3.6 mV) (Fig. 1c). We also calculated the median voltage of charge transfer (V_Median_, Eq. 3) for each data set (Fig. 1d). V_Median_ represents the membrane potential where the fully activated and fully resting states are equally populated^37^. This measure is distinct from the V_1/2_ because it allows for comparison between Boltzmann distributions that have an unequal number of transitions. The V_Median_ of deactivation, although roughly 10 mV more negative, was not statistically different compared to the V_Median_ of activation at either 22 °C or 36 °C (Fig. 1e, Supplemental Table S1). However, comparing the relative charge at each test potential did demonstrate that significantly less charge had returned during VSD deactivation at 22 °C (− 60 mV through − 10 mV) and 36 °C (− 60 mV through − 30 mV), compared with VSD activation (Fig. 1c). Despite their differences in their Boltzmann distributions, the V_Median_ values for activation and deactivation were comparable between recordings completed at 22 °C and 36 °C (Fig. 1e). Taken together, the distinct voltage dependence of VSD activation and deactivation demonstrate that, like at room temperature, VSD movement is hysteretic at physiological temperature and pulse durations consistent with the human ventricular action potential. These data also suggest that the process of VSD relaxation is intact at elevated temperatures.

Despite similarities in the voltage dependence of VSD movement, the kinetics of VSD movement at 22 °C and 36 °C displayed significant differences. We fitted gating current decay with an exponential function (Eq. 4) and observed that at physiological temperature gating currents elicited by depolarization from a − 100 mV holding potential (activation) displayed a single exponential rate of decay. Gating current decay at room temperature also displayed a single exponent of decay, but the time constants of decay were increased by an order of magnitude across all potentials measured (Fig. 1f). Gating current decay during VSD deactivation displays a second time constant that is significantly larger than the single time constant of decay observed during VSD activation, and indicative of the VSD’s transition from the relaxed state^12^. Upon deactivation from + 50 mV, the gating currents displayed a double exponential decay (Fig. 1f–h), consistent with the effects of VSD relaxation. Interestingly, the time course of current decay was unchanged between 22 and 36 °C (Fig. 1g), but the relative magnitude of the slow component of current decay was significantly reduced from 0.24 ± 0.04 to 0.08 ± 0.02 (Fig. 1h). These data highlight key differences in hERG 1 VSD movement between room temperature and near physiological temperature (36 °C). These data also provide, to our knowledge, the first characterization of hERG 1 voltage sensor behavior at physiological temperature.

### hERG 1a ionic current hysteresis at physiological temperature

**Figure 2:**
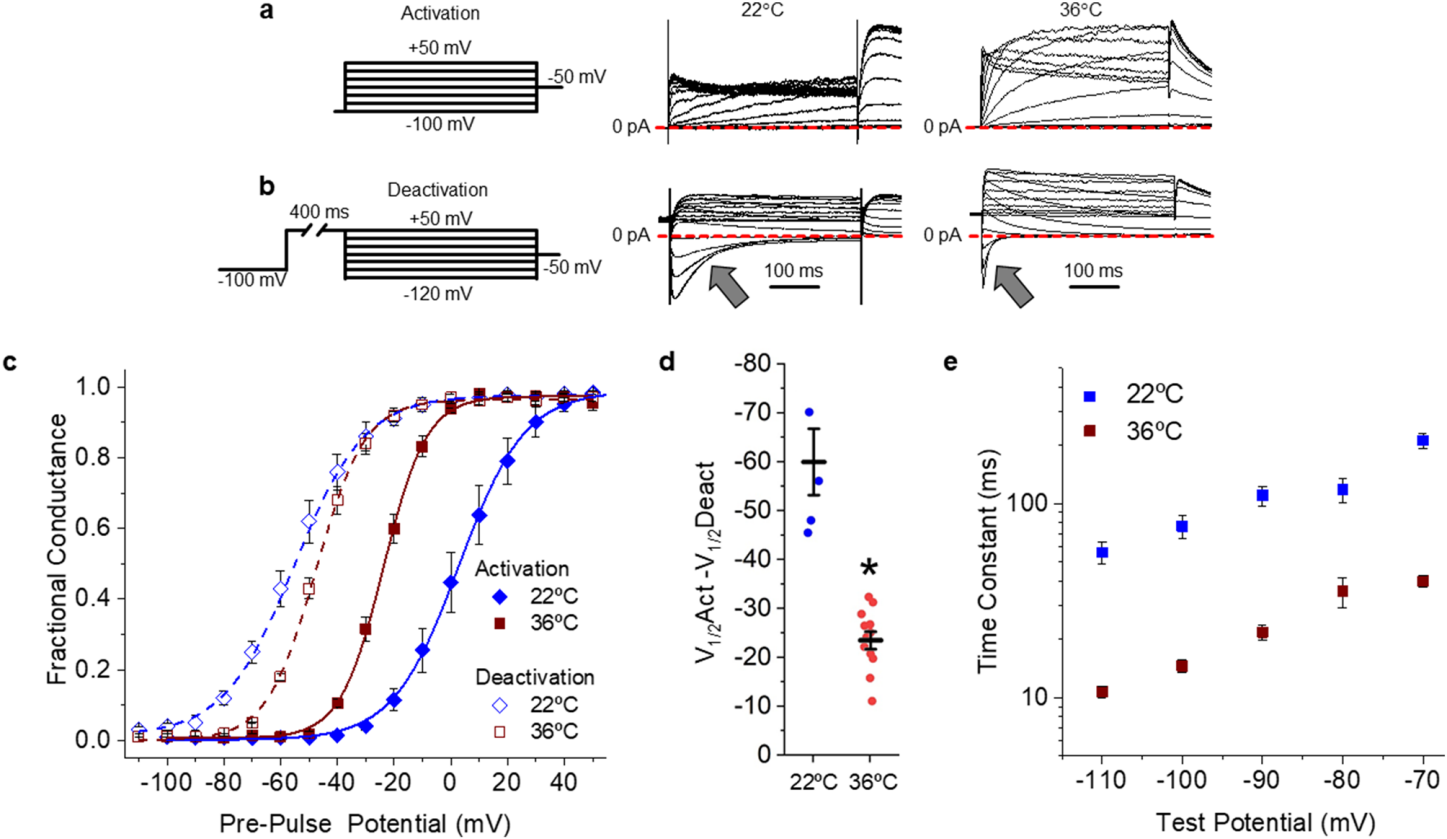
Physiological temperature reduces hERG 1a hysteresis of ionic currents. (**a**) Pulse protocol and corresponding sample ionic traces showing hERG 1a activation when stably expressed in HEK293 cells recorded at room temperature (22 °C, middle) or physiological temperature (36 °C, right). (**b**) Pulse protocol and corresponding sample ionic traces showing hERG 1a deactivation when stably expressed in HEK293 cells recorded at room temperature (22 °C, middle) or physiological temperature (36 °C, right). (**c**) Temperature-dependent shift in hERG 1a voltage dependence. Normalized peak tail current, representing fractional conductance, is plotted as a function of pre-pulse potential and fitted with a Boltzmann function for recordings completed at 22 °C (blue diamonds) and 36 °C (red squares). Channel activation recorded as in “(**a**)” is shown with solid symbols. Channel deactivation recorded as in “(**b**)” is shown with open symbols. (**d**) The magnitude of hysteresis was quantified by subtracting the V_1/2_ of deactivation from the V_1/2_ of activation. Hysteresis was significantly smaller in recordings completed at 36 °C (*n* = 12) compared with recordings at 22 °C (*n* = 5). (**e**) Time constants representing the fast deactivation time course for hERG 1a current recordings completed at either 22 °C (blue) or 36 °C (red). We fit current decay (arrows in “**b**”) with a double exponential equation (Eq. 4) and reported the fast time constant (τ) as a function of test potential. All data are reported as mean ± SEM. * indicates *p* < 0.05.

Ionic current also displays hysteretic behavior, where the voltage dependence of pore closure (deactivation) following a prolonged step to a positive potential is hyperpolarized relative to the voltage dependence of pore opening (activation)^13,15,16^. We sought to determine how the above reported hERG 1a VSD behavior translated to hERG 1a ionic currents. We recorded the voltage dependence of ionic current activation and deactivation using protocols identical in duration to those used to record gating charge. To assess the voltage dependence of activation we plotted hERG tail currents recorded at − 50 mV, as a function of pre-pulse potential and fitted those data with a Boltzmann function (Fig. 2a–c). At room temperature, hERG 1a currents displayed robust hysteresis, as expected, with a − 60.2 ± 6.8 (*n* = 5) mV difference between the voltage dependencies of activation and deactivation (Fig. 2c,d). At physiological temperature hERG activated at more negative potentials and deactivated at more positive potentials compared to room temperature recordings (Fig. 2c, Supplemental Table S2). The convergent shifts in voltage dependence at physiological temperature resulted in a significant reduction in the magnitude of hysteresis at physiological temperature (− 23.6 ± 1.8 mV, *n* = 12, *p* < 0.0001) compared with room temperature (Fig. 2c,d). Channel kinetics were also accelerated at physiological temperatures compared to room temperature (Fig. 2b,e). The accelerated activation and deactivation kinetics likely drive the concordant shifts in voltage dependence and reduced hysteresis at higher temperatures, as a greater number of channels are able to transition between states during the relatively short (400 ms) conditioning pulses. These data demonstrate that hysteresis of hERG ionic current is intact, but notably reduced, under physiologically relevant conditions.

### hERG 1a/1b gating charge at physiological temperature

**Figure 3:**
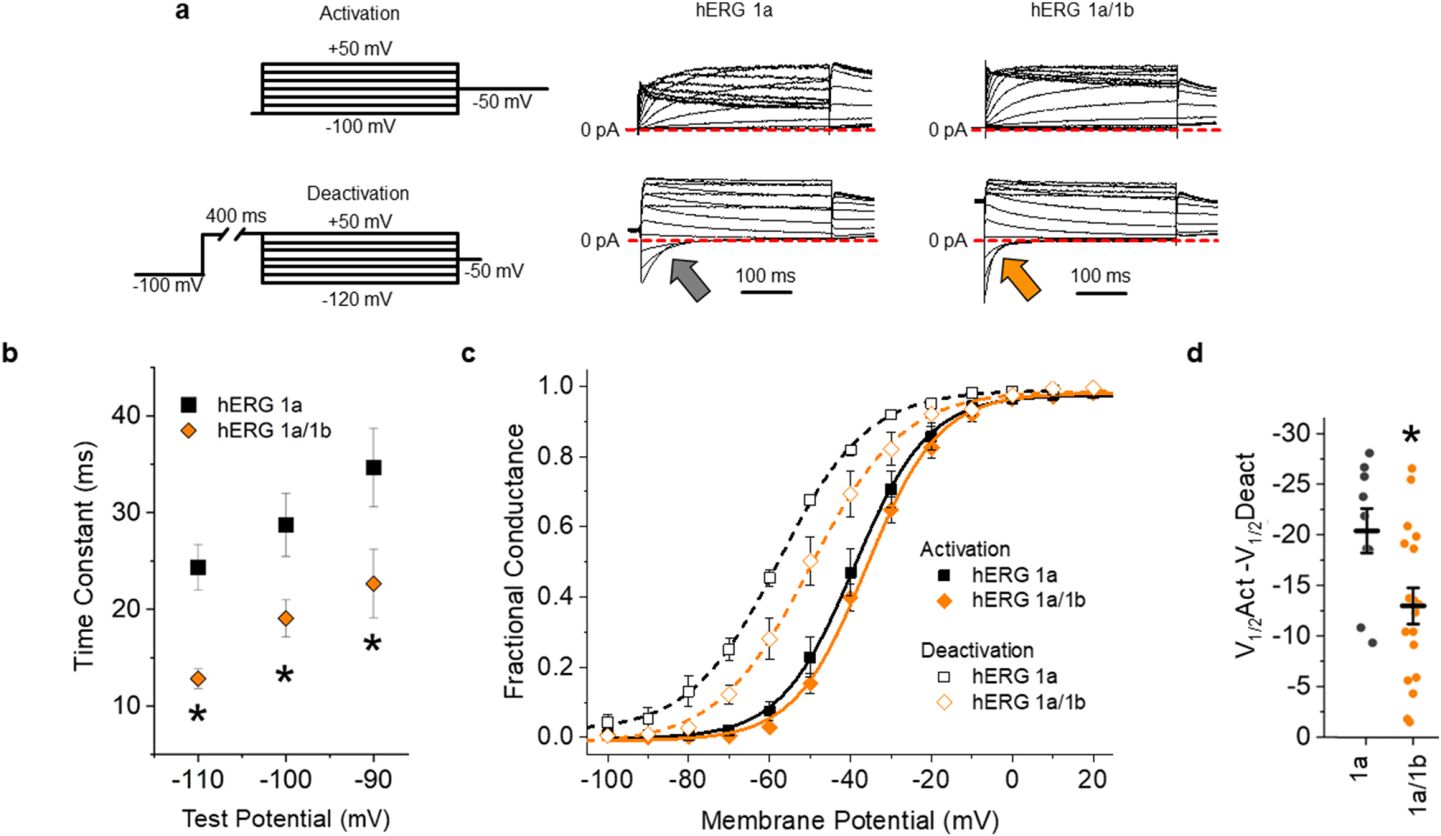
hERG 1b expression reduces hERG channel hysteresis of ionic current. (**a**) Pulse protocol and corresponding sample ionic traces showing hERG 1a (middle) and hERG 1a/1b channel (right) activation when stably expressed in HEK293 cells recorded at physiological temperature (36 °C). (**b**) Pulse protocol and corresponding sample ionic traces showing hERG 1a (middle) and hERG 1a/1b channel (right) deactivation when stably expressed in HEK293 cells recorded at physiological temperature (36 °C). (**c**) hERG 1b modifies hERG channel voltage dependence. Normalized peak tail current, representing fractional conductance, is plotted as a function of pre-pulse potential and fitted with a Boltzmann function for hERG 1a (black) and hERG 1a/1b (orange). Channel activation recorded as in “(**a**)” is shown with solid symbols. Channel deactivation recorded as in “(**a**)” is shown with open symbols. (**d**) The magnitude of hysteresis was quantified by subtracting the V_1/2_ of deactivation from the V_1/2_ of activation. Hysteresis was significantly smaller in hERG 1a/1b heteromeric channels (*n* = 10) compared with hERG 1a homomeric channels (*n* = 7). All data are reported as mean ± SEM. * indicates *p* < 0.05.

Native hERG channels are heteromeric, containing both hERG 1a and hERG 1b subunits, and thus have subunits with (hERG 1a) and without (hERG 1b) a functional PAS domain^17,18,38,39^. Previous studies completed at room temperature demonstrated that PAS disruption accelerates VSD movement^13,15,16^. These studies suggest that heteromeric hERG 1a/1b channels, comprising PAS-deficient hERG 1b subunits, would display accelerated VSD movement and reduced hysteresis compared to hERG 1a homomeric channels. To test this hypothesis, we repeated the experiments above using a cell line constitutively expressing hERG 1a, but with hERG 1b under control of an inducible promoter (see “Methods”^40^) (Fig. 3).

We treated inducible hERG 1b cells with 100 μg/mL doxycycline 48 h prior to recording and validated hERG 1b induction by recording the time course of ionic current deactivation (Fig. 3a,b). 100 μg/mL doxycycline significantly accelerated hERG current deactivation, demonstrating that the hERG 1b subunit was successfully co-expressed with hERG 1a^40^. Induced hERG 1b expression with doxycycline also depolarized the voltage dependence of deactivation without affecting the voltage dependence of activation (Fig. 3c, Supplemental Table S3), which significantly reduced the magnitude of hysteresis of ionic current (Fig. 3d). These data are consistent with other reports of the impact of hERG 1b on hERG 1 channel gating^34,35,41,42^, and demonstrate that hERG 1b expression was successfully induced.

**Figure 4:**
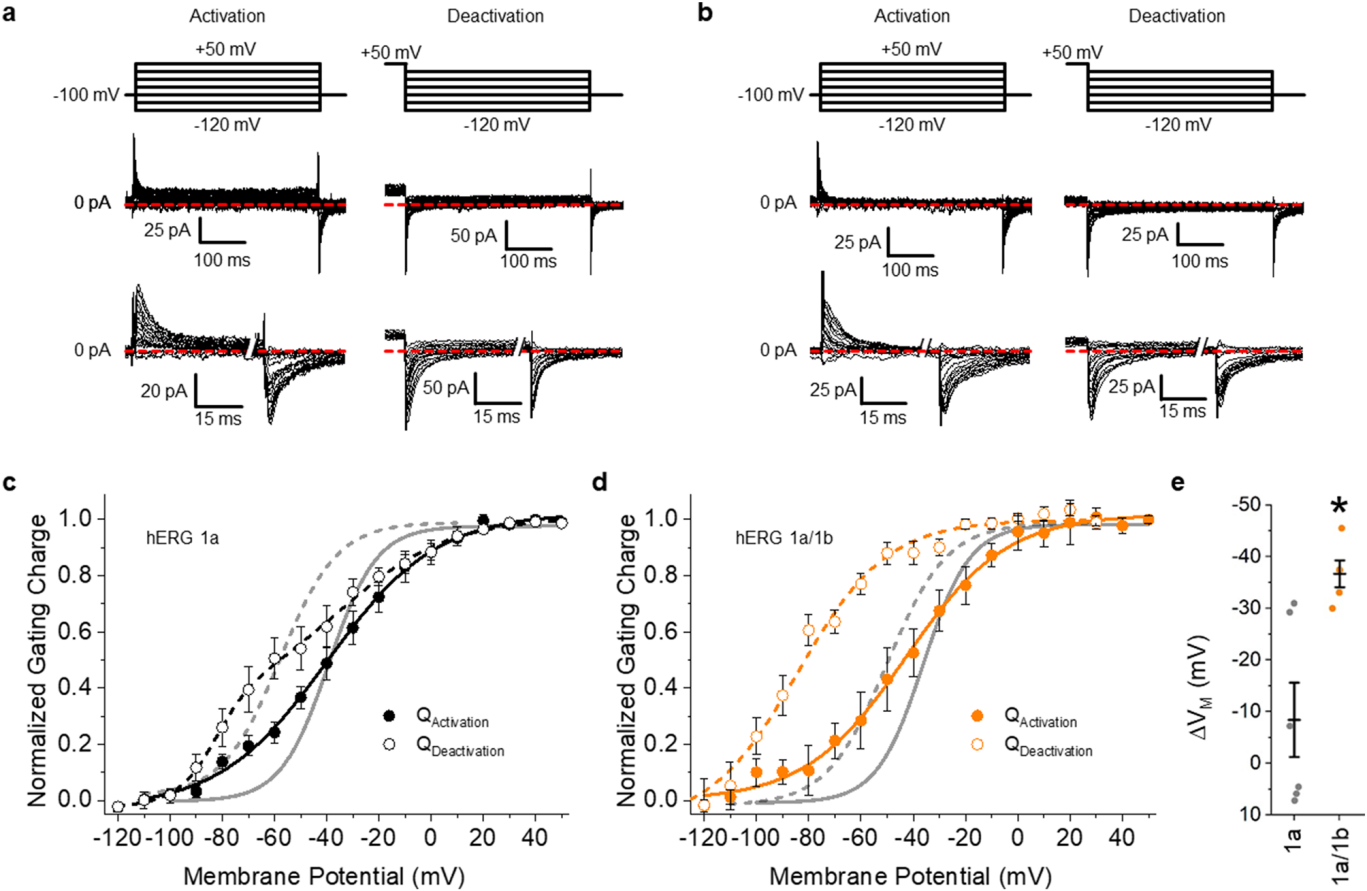
hERG 1b expression transforms hERG channel gating charge. (**a**) Activation and deactivation pulse protocols (top) and corresponding activation and deactivation gating current traces recorded at 36 ± 1 °C from HEK293 cells stably expressing hERG 1a. An expanded view of the gating current traces is show at bottom. (**b**) Activation and deactivation pulse protocols (top) and corresponding activation and deactivation gating current traces recorded at 36 ± 1 °C from HEK293 cells stably expressing hERG 1a and hERG 1b. An expanded view of the gating current traces is show at bottom. (**c**) Voltage dependence of hERG 1a gating charge activation (filled circles) and deactivation (open circles). Gating currents elicited during the test pulse were integrated, and the resultant gating charge was normalized, plotted as a function of test potential and fitted with a Boltzmann function. Voltage dependence of ionic current activation and deactivation are shown in gray. (**d**) Voltage dependence of hERG 1a/1b gating charge activation (filled circles) and deactivation (open circles). Voltage dependence of ionic current activation and deactivation are shown in gray. Expression of hERG 1b with hERG 1a triggered disparate shifts in the voltage dependence of VSD and ionic deactivation. (**e**) The magnitude of hysteresis was quantified by subtracting the V_median_ of deactivation from the V_median_ of activation. Hysteresis was significantly larger when hERG 1b was coexpressed with hERG 1a (*n* = 10) compared with hERG 1a expressed alone (*n* = 8). All data are reported as mean ± SEM. * indicates *p* < 0.05.

Inducing hERG 1b expression triggered marked and unexpected changes in voltage sensor deactivation, but not activation (Fig. 4). hERG 1b transformed the double Boltzmann distribution generated from hERG 1a alone (Figs. 1c, 4c) into a single Boltzmann (Fig. 4d), (V_Median_: − 66.7 ± 5.3 mV, *p* < 0.05, Supplemental Table S4), and increased the hysteresis between VSD activation and deactivation (Fig. 4e). hERG 1b induction did not affect the voltage dependence of activation (Supplemental Table S4). Consequently, the magnitude of hysteresis between voltage sensor activation and deactivation, reported as the difference in V_Median_, was significantly larger in cells expressing hERG 1b (Fig. 4e, Supplemental Table S4). The leftward shift of the V_Median_ of deactivation in heteromeric channels is in opposition with the effects observed on deactivation of ion current, implying that hERG 1b alters the communication between the VSD and pore. These data are the first recordings, to our knowledge, of gating charge from heteromeric hERG 1a/1b channels. These data also show that hERG 1b enhances VSD hysteresis while altering voltage sensor and pore coupling during deactivation.

### PAS action determines VSD movement characteristics

Induced hERG 1b expression with hERG 1a hyperpolarized voltage dependence of gating charge deactivation and depolarized the voltage dependence of ionic current deactivation compared to homomeric hERG 1a channels. As hERG 1b and hERG 1a differ only in their N-terminal domains, the reduced hERG 1a PAS domain abundance in heteromeric channels stands out as the most likely culprit underlying the altered VSD behavior in hERG 1a/1b channels compared to hERG 1a channels.

To determine if the relative activity of the hERG 1a PAS domain is driving the differences in hERG 1a and hERG 1a/1b VSD movement, we characterized the effect of targeted PAS disruption on hERG 1a homomeric channel VSD movement using PAS domain-targeting single chain variable fragment (scFv) antibodies, scFv2.10 and scFv2.12^43^. scFv2.10 and scF2.12 bind and disrupt PAS action, and thereby induce a heteromeric hERG 1a/1b-like phenotype upon ionic currents recorded from homomeric hERG 1a channels^43,44^. We therefore hypothesized that PAS disruption with the scFv antibodies would transform hERG 1a gating currents into a phenotype similar to hERG 1a/1b gating currents.

**Figure 5:**
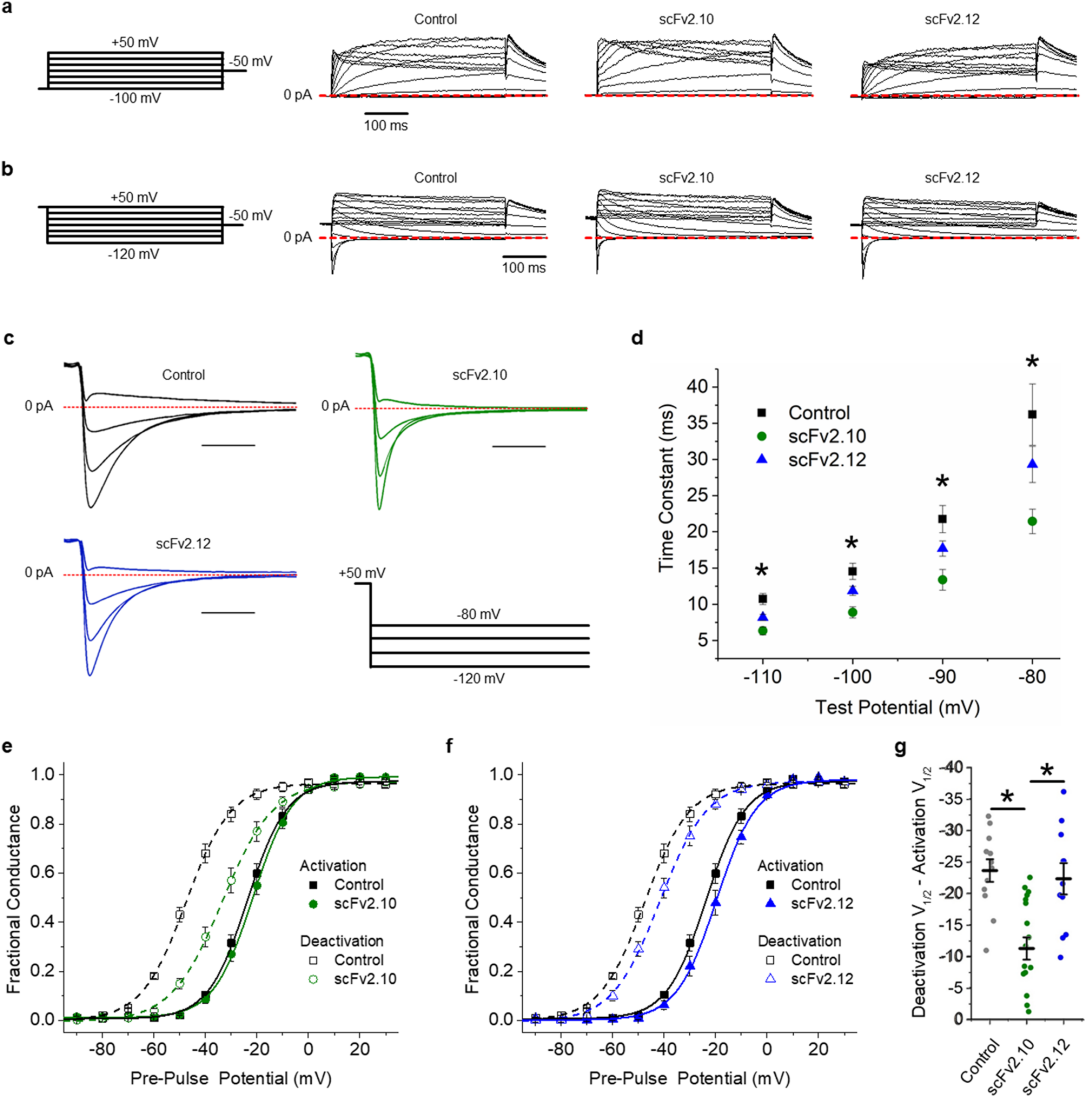
Effects of anti-PAS antibodies on hERG 1a ionic current hysteresis. (**a**) Pulse protocol (left) and corresponding sample ionic traces showing hERG 1a activation in vehicle control solution or in the presence of 10 µM scFv2.10 or 10 µM scFv2.12. (**b**) Pulse protocol (left) and corresponding sample ionic traces showing hERG 1a deactivation in vehicle control solution or in the presence of scFv2.10 or scFv2.12. (**c**) scFv2.10 modification of hERG 1a voltage dependence. Normalized peak tail current, representing fractional conductance, is plotted as a function of pre-pulse potential and fitted with a Boltzmann function for hERG 1a recordings in vehicle control solution (black) and scFv2.10 (green). (**d**) scFv2.12 modification of hERG 1a voltage dependence. Normalized peak tail current, representing fractional conductance, is plotted as a function of pre-pulse potential and fitted with a Boltzmann function for hERG 1a recordings in vehicle control solution (black) and scFv2.12 (blue). (**e**) The magnitude of hysteresis was quantified by subtracting the V_1/2_ of deactivation from the V_1/2_ of activation. Hysteresis was significantly smaller in the presence of scFv2.10 (*n* = 14) compared with control recordings (*n* = 12) and recordings in the presence of scFv2.12 (*n* = 6). (**f**) Expanded view of deactivating hERG 1a ionic currents recorded from Control (black), scFv2.10 (green) and scFv2.12 (blue) cells. (**g**) Fast time constants of deactivation plotted as a function of test potential for Control (black), scFv2.10 (green) and scFv2.12 (blue) cells. Time constants were derived by fitting current decay with a double exponential function. All data are reported as mean ± SEM. * indicates *p* < 0.05.

We validated scFv-mediated PAS disruption by recording hERG 1a ionic currents in the presence of each scFv antibody delivered intracellularly through the recording pipette (Fig. 5). Like hERG 1b, scFv2.10 and scFv2.12 both accelerated the deactivation time course (Fig. 5c,d,g), which we measured by fitting current decay with an exponential function (Eq. 4). The accelerated deactivation time course in the presence of scFv2.10 and scFv2.12 also corresponded with concordant rightward shifts in the voltage dependence of deactivation (Fig. 5e,f, Supplemental Table S1). Despite the mutual shifts in the voltage dependence of deactivation, only scFv2.10 significantly reduced the magnitude of the hysteresis between activation and deactivation (Fig. 5e).

**Figure 6:**
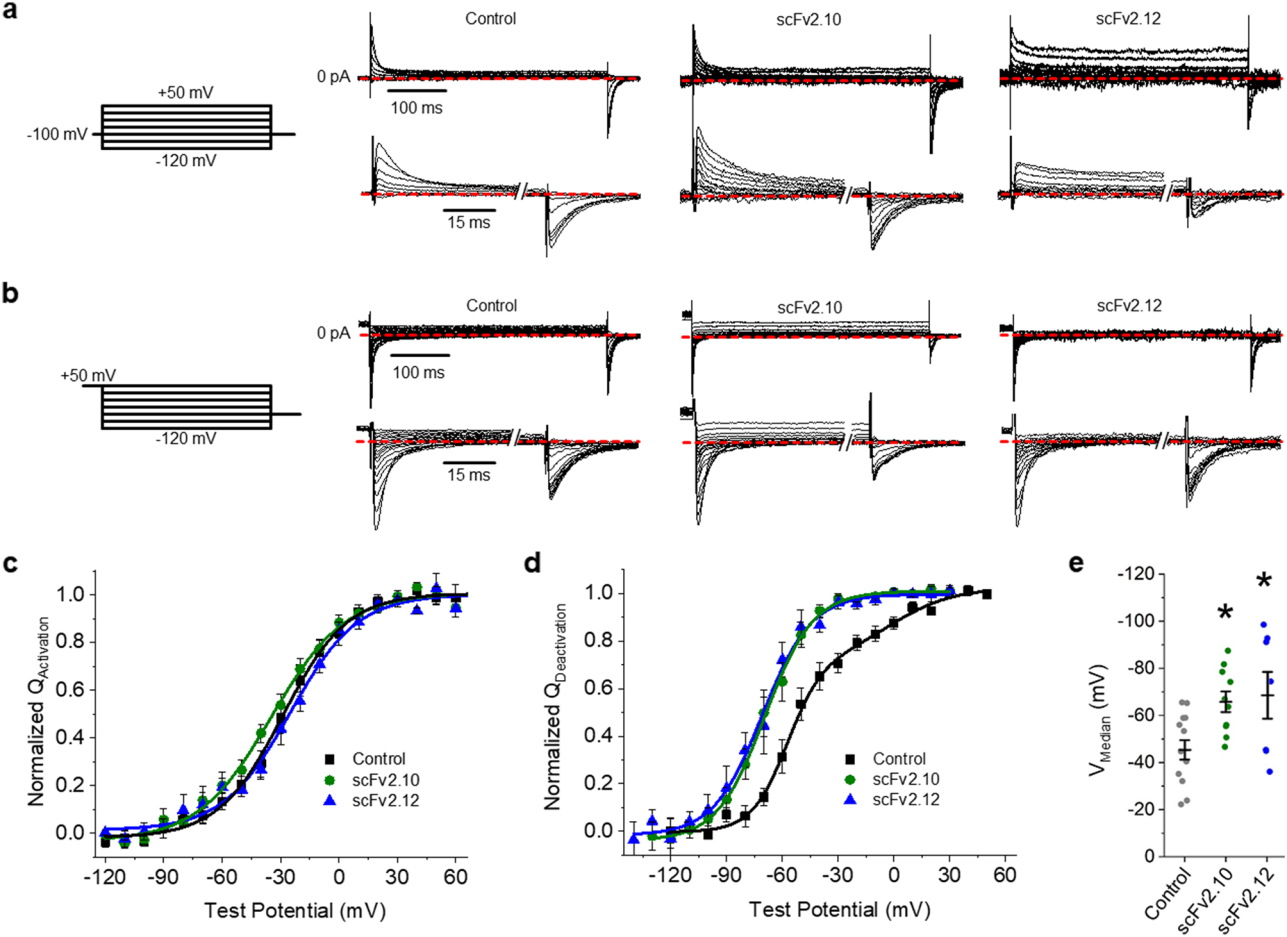
Anti-PAS antibodies transform hERG 1a gating charge. (**a**) The pulse protocol (left) and corresponding activation gating current traces for vehicle controls and in the presence of either 10 µM scFv2.10 (middle right) or 10 µM scFv2.12 (right). hERG 1a gating charge activation was measured by recording gating currents elicited by depolarization from a negative (− 100 mV) holding potential. (**b**) The pulse protocol (left) and corresponding deactivation gating current traces for vehicle controls and in the presence of either 10 µM scFv2.10 or 10 µM scFv2.12. hERG 1a gating charge deactivation was measured by recording gating currents elicited by hyperpolarization from a positive (+ 50 mV) holding potential. (**c**) Voltage dependence of hERG 1a voltage sensor activation from vehicle controls (black squares) or in the presence of either 10 µM scFv2.10 (green circles) or 10 µM scFv2.12 (blue triangles). Gating currents elicited during the test pulse were integrated, and the resultant gating charge was normalized, plotted as a function of test potential, and fitted with a Boltzmann function. (**d**) scFv antibodies hyperpolarize voltage sensor deactivation. Voltage dependence of hERG 1a voltage sensor deactivation from vehicle controls (black squares) or in the presence of either 10 µM scFv2.10 (green circles) or 10 µM scFv2.12 (blue triangles). Gating currents elicited during the test pulse were integrated, and the resultant gating charge was normalized, plotted as a function of test potential, and fitted with either a single Boltzmann (scFv2.10 and scFv2.12) or a double Boltzmann (control) function. (**e**) Bar graph depicting the mean V_median_ of deactivation for control (*n* = 12), scFv2.10 (*n* = 14), and scFv2.12 (*n* = 6). Both scFv antibodies significantly hyperpolarized the V_median_ of VSD deactivation. All data are reported as mean ± SEM. * indicates *p* < 0.05.

**Figure 7:**
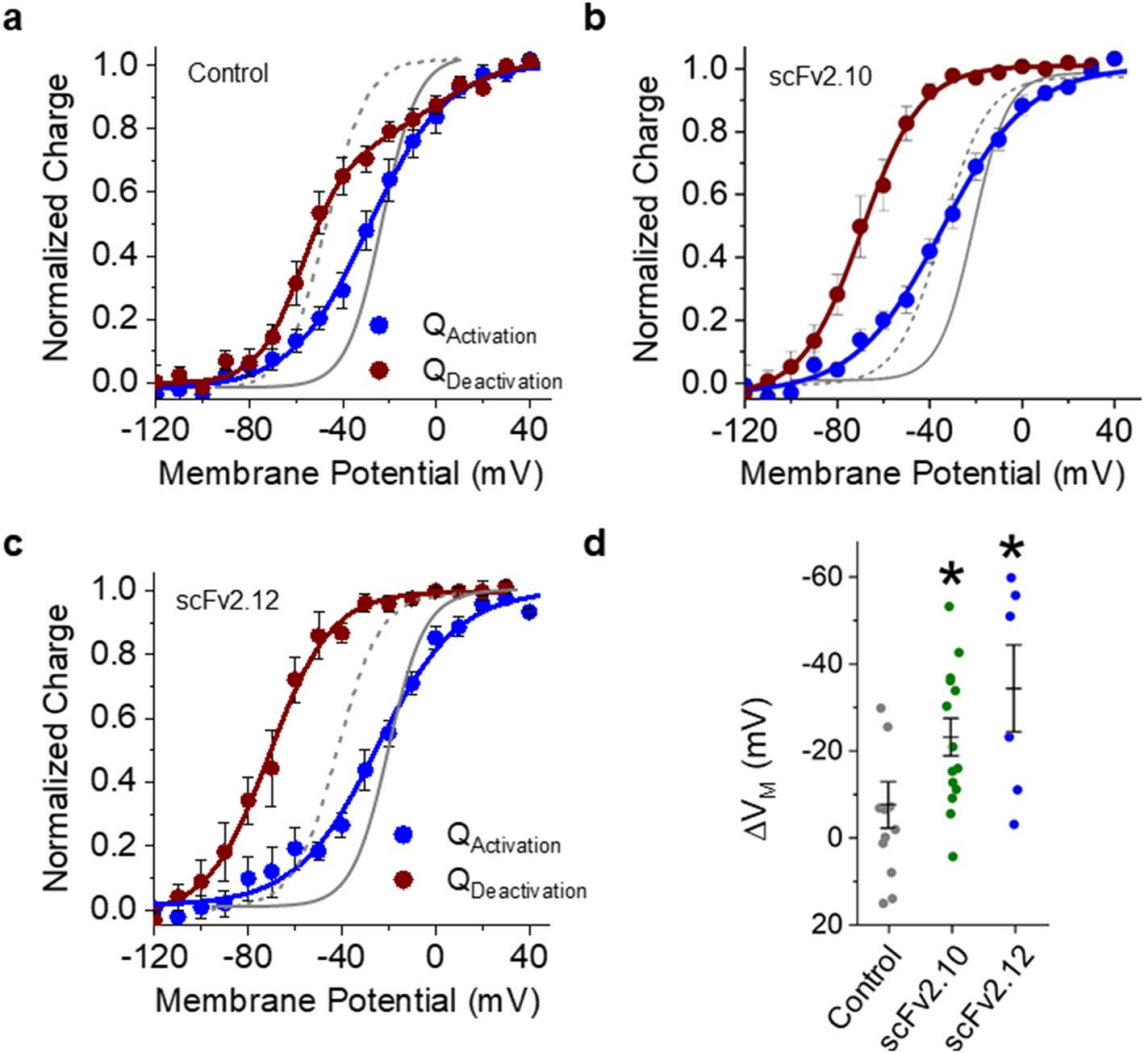
Anti-PAS antibodies enhance VSD hysteresis. (a-c) Voltage dependence of hERG 1a voltage sensor activation (blue) and deactivation (red) for vehicle controls (**a**), scFv2.10 (**b**), and scFv2.12 (**c**). Fits from ionic currents are shown in gray. Gating currents elicited during the test pulse were integrated, and the resultant gating charge was normalized, plotted as a function of test potential and fitted with a Boltzmann function. (**d**) The magnitude of hysteresis was quantified by subtracting the V_median_ of deactivation from the V_median_ of activation. Hysteresis was significantly larger in the presence of scFv2.10 (*n* = 14) or the presence of scFv2.12 (*n* = 6) compared with vehicle control recordings (*n* = 12). All data are reported as mean ± SEM. * indicates *p* < 0.05.

Next, we recorded gating currents at 36 ± 1 °C with either scFv2.10 or scFv2.12 (Fig. 6). Similar to the effects of hERG 1b, both antibodies transformed the double Boltzmann distribution observed under control conditions into a single Boltzmann (Fig. 6b,d), with V_Median_ values of − 66.1 ± 4.4 mV and − 69.0 ± 10.0 mV, respectively (Fig. 6e, Supplemental Table S1). Neither antibody affected voltage sensor activation (Fig. 6a,c). Consequently, the hysteresis of gating charge between activation and deactivation was significantly larger in the presence of either PAS-targeting antibody (Fig. 7, Supplemental Table S1). These data demonstrate that PAS action underlies the observed differences in homomeric hERG 1a and heteromeric hERG1a/1b VSD movement.

## Discussion

Here we sought to measure hERG 1 gating charge at physiological temperatures. In doing so, we identified differences in VSD behavior between hERG 1a currents recorded at 22 °C and 36 °C, as well as a fundamental difference in VSD behavior between homomeric hERG 1a and heteromeric hERG 1a/1b channels. Physiological temperature uniquely accelerated gating current decay during activating (reduced time constant) pulses compared to deactivating pulses (reduced slow component of decay). We also found that hERG 1a voltage sensor movement is hysteretic at near physiological temperature (36 °C). Gating currents elicited by depolarization from a negative holding potential (VSD activation) display a single time constant of decay as well as a single Boltzmann distribution. Gating currents elicited by hyperpolarization from a positive potential (VSD deactivation) displayed two time constants of decay and a double Boltzmann distribution. These data demonstrate that VSD relaxation is intact in hERG channels at physiological temperature. Additionally, disrupting PAS function by either induced hERG 1b expression or PAS-targeting scFvs transformed the double Boltzmann distribution into a single Boltzmann distribution, and hyperpolarized the voltage dependence of VSD deactivation.

Remarkably, disrupting the PAS domain had an apparent opposing effect at the pore gate, where the voltage dependence of ionic current deactivation was depolarized in the presence of hERG 1b or the scFv antibodies compared to hERG 1a alone. The shift in the ionic deactivation could be largely attributed to an accelerated time course of deactivation and therefore better capture of the voltage dependence of deactivation at the chosen step duration, instead of an impact on the energetics of pore closure. However, it still stands that the VSD is becoming less likely to return, while the pore is closing faster. The disparate effects on the VSD and pore could suggest that PAS disruption uncouples the voltage sensor from the pore during deactivation.

In this study we provide, to our knowledge, the first report of hERG channel gating charge at physiological temperature. From these recordings there where two key findings. First, VSD movement is hysteretic at physiological temperatures. Delayed hERG pore closure has been suggested to play a cardioprotective role against arrhythmia by prolonging the effective refractory period^45,46^. Our recordings were completed using pulse durations comparable to the human ventricular action potential (400 ms). These data therefore demonstrate that hERG voltage sensor hysteresis, indicative of relaxation, is one mechanism by which pore closure is slowed to enhance hERG channel open probability at the end of the cardiac action potential. Second, VSD deactivation displayed a double Boltzmann distribution, but only from unperturbed hERG 1a homomeric channels. A previous study at room temperature in tsA201 cells reported two charge centers in the hERG VSD that generated a double Boltzmann distribution during VSD activation but not deactivation^36^. The differences in the presentation of the double Boltzmann functions of our study at physiological temperature and the room temperature study could indicate that the charge centers of hERG 1 have distinct Q_10_ values. Two charge systems were also observed in *Shaker* and K_V_1.5 channels^47,48^. Our study supports the finding that two charge centers regulate hERG channel gating and expands upon this to demonstrate that the voltage dependencies of these charge centers are distinctly regulated by the PAS domain. We also postulate that the more negative transition (≈ − 53 mV) represents recovery from the relaxed state, and the less negative transition (≈ − 21 mV) is unrelated to VSD relaxation.

Native cardiac I_Kr_ is conducted by a mixed population homomeric and heteromeric 1a/1b channels^17,18,34^. hERG 1b has a unique, PAS deficient N-terminus, that accelerates the kinetics and enhances the magnitude of ionic current conducted by heteromeric hERG 1a/1b channels compared to homomeric hERG 1a channels^34,41^. We characterized gating charge recorded from heteromeric channels to generate mechanistic insight into heteromeric channel ionic currents. When we induced hERG 1b expression upon hERG 1a expressing cells the resulting heteromeric channel gating currents where nearly identical to currents recorded from scFv-bound hERG 1a channels. The phenotype of ionic currents recorded from heteromeric 1a/1b channels and scFv-bound hERG1a channels is also strikingly similar to that observed in channels carrying PAS domain mutations. The most notable of these is R56Q which imparts a heteromeric channel-like gating phenotype without affecting channel trafficking^49,50^. The R56Q mutant accelerates hERG 1a channel gating by disrupting a salt bridge at the interface between the N-terminal PAS and C-terminal CNBHD domains^27^, abolishing the PAS/CNBHD interaction^31^, and effectively disabling the hERG 1a PAS domain. We postulate that scFv2.10/scFv2.12 binding with the hERG 1a PAS domain disrupts the PAS/CNBHD interaction, effectively disabling the PAS domain as a channel modulator. In vivo, where the relative hERG 1a and hERG 1b levels change developmentally^51^ and pathophysiologically^52^, we predict that the stoichiometric relationship of hERG 1a and hERG 1b is used to fine tune I_Kr_ to meet cardiac demand.

Finally, in two previous studies, scFv2.10 and scFv2.12 were shown to bind the hERG PAS domain and presumably disrupt its ability to regulate channel gating, inducing a PAS-deficient behavior on hERG ionic currents^43,44^. Here we demonstrate that scFv2.10 and scFv2.12, by disrupting PAS action, manipulate hERG VSD movement and its coupling with the pore. Several studies at room temperature have demonstrated that the hERG 1a PAS domain slows VSD movement and enhances gating current hysteresis^13,15^. Those studies also reported concomitant changes in the voltage dependence of ionic currents. More recently, an apparent uncoupling between the hERG VSD and pore gate was reported during prolonged depolarization^16^. In that study, the authors reported that while the voltage dependence of the VSD and pore activation progressively converged with prolonged depolarization, the voltage dependence of VSD and pore deactivation became increasingly disparate^16^. Our study appears to contrast many of these prior findings in that in our hands PAS disruption appeared to simultaneously promote VSD hysteresis and uncouple VSD movement from pore closure during deactivation. We presume that these disparate findings could be consequent to the recording temperatures.

## Summary

The data presented here demonstrate that the hERG 1a and hERG 1a/1b VSD movement is hysteretic at near physiological temperature and time scales. We also identified that PAS action from the hERG 1a subunit underlies the fundamental differences in VSD deactivation observed in homomeric hERG 1a and heteromeric hERG 1a/1b channels.

## Materials and methods

### Cell culture

HEK293 cells were cultured in minimum essential medium (MEM, Invitrogen, Cat. No. 11095080) supplemented with 10% fetal bovine serum (Thermo Fisher, Cat. No. SH30070.03). HEK293 cells stably expressing the hERG 1a subunit alone were cultured in MEM supplemented with 5 μg/mL of G418 (Thermo Fisher, Cat. No. 11811031). HEK293 cells stably co-expressing hERG 1a and hERG 1b were cultured in MEM medium supplemented with 5 μg/mL of G418 and 0.25 μg/mL of puromycin (Clontech, Cat. 631306). hERG 1b was expressed on demand, using the Tet-One system by Takara (Clontech, Cat. No. 634301). We induced hERG 1b protein expression using 100 μg/mL doxycycline 48 h prior to recording. Details on the development and maintenance of this cell line were published previously^40^.

### Electrophysiology

All recordings were completed at either room temperature (22 °C) or near physiological temperature (36 ± 1 °C) using whole-cell patch clamp with an Axon 200A amplifier and Clampex (Molecular Devices). Leak subtraction was performed off-line based on measured current observed at potentials negative to hERG 1 activation. The inter-pulse duration for all recordings was 10 s.

### Ionic recordings

Data were sampled at 10 kHz and low-pass filtered at 1 kHz. Cells were perfused with extracellular solution containing (in mM): 150 NaCl, 5.4 KCl, 1.8 CaCl_2_, 1 MgCl_2_, 15 glucose, 10 HEPES, 1 Na-pyruvate, and titrated to pH 7.4 using NaOH. Recording pipettes had resistances of 2–4.5 MΩ when backfilled with intracellular solution containing (in mM): 5 NaCl, 150 KCl, 2 CaCl_2_, 5 EGTA, 10 HEPES, 5 MgATP and titrated to pH 7.2 using KOH. We supplemented the intracellular solution with scFv antibodies at 10 μM to measure the effects of the antibodies. Intracellular solution aliquots were kept frozen until the day of recording. The intracellular solution was kept on ice during recordings and discarded 2–3 h post-thaw.

To assess the voltage dependence of hERG 1 activation, cells were stepped from a − 100 mV holding potential to a 400 ms pre-pulse between − 100 and + 50 mV in 10 mV increments. Tail currents were then measured during a − 50 mV, 3 s test pulse. To assess the voltage dependence of hERG 1 deactivation, cells were stepped from a − 100 mV holding potential to a + 50 mV conditioning pulse for 400 ms before stepping to a 400 ms pre-pulse potential between + 50 and − 110 mV. Tail currents were then measured during a − 50 mV, 3 s test pulse. To describe the voltage dependence of activation or deactivation, peak tail current was normalized to cellular capacitance, plotted as a function of pre-pulse potential, and fitted with the following Boltzmann equation:

1 $$y = \left[ {\frac{{A_{1} - A_{2} }}{{1 + e^{{( {V - V_{0} } )/k}} }}} \right] + A_{2} ,$$

where *A*_1_ and *A*_2_ represent the maximum and minimums of the fit, respectively, *V* is the membrane potential, *V*_0_ is the midpoint, and k is the slope factor. Only recordings that maintained a series resistance at or below 10 MΩ were used for ionic recordings. Series resistance was compensated up to 50%, generating a voltage error of < 4 mV.

### Gating currents

Data were sampled at 20 kHz and low-pass filtered at 10 kHz. Cells were perfused with extracellular solution containing (in mM): 140 NMDG, 10 HEPES, 10 dextrose, 1 MgCl_2_, 1 CaCl_2_, and titrated to pH 7.4 using HCl. Recording pipettes had resistances of 4–7.5 MΩ when backfilled with intracellular solution containing (in mM): 140 TEA-OH, 1 MgCl_2_, 10 EGTA, and 10 HEPES, adjusted to pH 7.2 using HF. We supplemented the intracellular solution with scFv antibodies at 10 μM to measure the effects of the antibodies. The intracellular solution was kept on ice during recordings.

Following membrane rupture, cells were maintained at − 100 mV for 5 min to allow the pipette solution to fully diffuse into the cell. Capacitance transients were compensated manually at − 120 mV using a + 20 mV test pulse at maximal gain. The capacitance transient was checked after each recording to ensure that it had remained constant. We discarded recordings where the capacitance transient compensation did not remain constant. Only cells expressing hERG 1 displayed voltage-dependent capacitive currents (Supplementary Fig. S1). To assess the voltage dependence of hERG 1 voltage sensor activation, cells were stepped from a − 100 mV holding potential to a 400 ms pulse between − 120 and + 50 mV in 10 mV increments. To assess the voltage dependence of hERG 1 voltage sensor deactivation, cells were stepped from a − 100 mV holding potential to a + 50 mV conditioning pulse for 400 ms before stepping to a 400 ms pre-pulse potential between + 50 and − 120 mV.

To describe the voltage dependence of voltage sensor activation or deactivation, the integral of gating currents elicited during each test pulse were plotted as a function of test potential, and fitted with either a single Boltzmann equation (Eq. 1) or a double Boltzmann equation:

2 $$y = \left[ {\left( {\frac{{A_{1} }}{{1 + e^{{\left( {V - V_{1} } \right)/k_{1} }} }}} \right) + \left( {\frac{{A_{2} }}{{1 + e^{{\left( {V - V_{2} } \right)/k_{2} }} }}} \right)} \right] + A_{3} ,$$

where *A*_1_ is the relative amplitude of transition 1, *A*_2_ is the relative amplitude of transition 2, *A*_3_ is the minimum of the fit, *V* is the membrane potential, *V*_1_ and *V*_2_ are the respective midpoints of each transition, and *k*_1_ and *k*_2_ are the respective slope factors of each transition. We derived the V_Median_ for each charge versus voltage (Q/V) curve by measuring the area between the curve and the ordinate (Q) axis as described by Chowdhury and Chanda^37^, where the area between the curve and the Q axis is calculated using the trapezoid method:

3 $$V_{Median} = \frac{1}{2}\mathop \sum \limits_{i = 1}^{n - 1} \left( {V_{i + 1} + V_{i} } \right)\left( {Q_{i + 1} - Q_{i} } \right),$$

where *V*_*i*_ is the *i*th point on the QV curve and *Q*_*i*_ is the fraction of charge that is transferred at *V*_*i*_.

Ionic and gating current kinetics were assessed by fitting current decay during the test pulse with a single or a double exponential function:

4 $$y = Y_{0} + A_{1} e^{{ - t/\tau_{1} }} + A_{2} e^{{ - t/\tau_{2} }} ,$$

where *Y*_0_ is the asymptote, *A*_1_ and *A*_2_ are the relative components of the fast and slow time constants *τ*_1_ and *τ*_2_, respectively. Only gating current recordings that maintained series resistance below 20 MΩ were used. Series resistance was compensated up to 50% generating a voltage error of ~ 1 mV.

### Statistical analysis

Analysis was completed using Clampfit (Molecular Devices) and Origin (OriginLab). All data are reported as mean ± SEM and were compared using a Student’s *t* tests. When applicable, an ANOVA and Bonferroni post hoc *t* tests where used. Statistical significance was taken at *p* < 0.05. Data points greater than two times the standard deviation were termed outliers and excluded from analysis.

## Supplementary Information

Supplementary Information.

## Data Availability

The datasets generated during and/or analyzed during the current study are available from the corresponding author on reasonable request.
