# Supplementary material for: Hysteretic hERG channel gating current recorded at physiological temperature
Source: Sci Rep. 2022 Apr 8;12:5950. doi: 10.1038/s41598-022-10003-7 (PMC8993916; doi:10.1038/s41598-022-10003-7)
Supplement: Supplementary file 1 — Supplementary Information. [file 41598_2022_10003_MOESM1_ESM.pdf]

Supplemental Material for:

Hysteretic hERG channel gating current recorded at physiological temperature

David K. Jones[1,2]

[1]Department of Pharmacology
University of Michigan School of Medicine
Ann Arbor, MI 48109

[2]Department of Internal Medicine
University of Michigan School of Medicine
Ann Arbor, MI 48109

Correspondence: davekj@umich.edu

Supplementary Figure S1

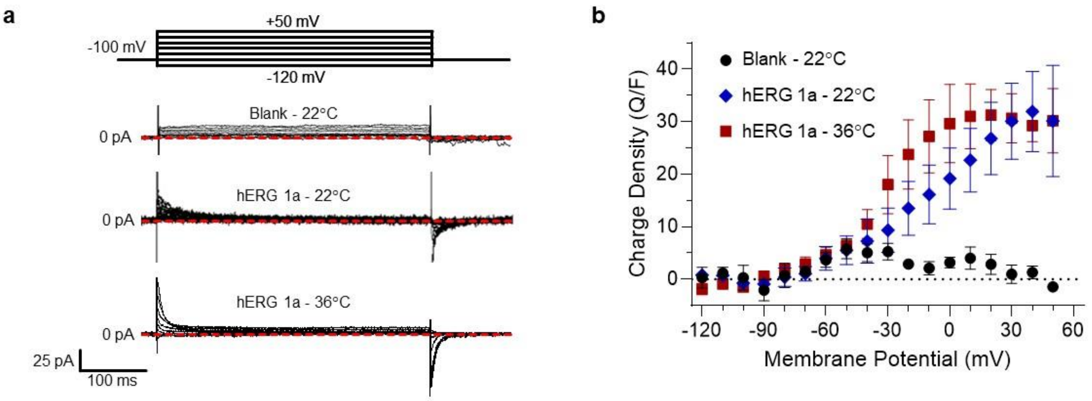

Supplementary Figure S1. Charge density recorded from blank HEK293 cells and HEK293 cells stably expressing hERG 1a. (**a**) Sample current traces recorded at 22 °C from blank HEK293 cells (*top*) or HEK293 cells expressing hERG 1a recorded at 22 °C (*middle*) and 36 °C (*bottom*). Membrane capacitance transients were compensated from a + 20 mV depolarizing pulse from a -120 mV holding potential. Following compensation, currents elicited during the step pulse of the pulse protocol shown in "a" were integrated, normalized to membrane capacitance, and plotted as a function of test potential. (**b**) Quantification of recordings as shown in "a" for blank HEK293 cells (*black circles*) and HEK293 cells expressing hERG 1a recorded at 22 °C (*blue diamonds*) and 36 °C (*red squares*). Only HEK293 cells expressing hERG 1a displayed measurable gating charge. All data are reported as mean ± SEM. n = 6 - 12

Supplementary Tables

Supplemental Table S1. hERG 1a Gating Charge Activation & Deactivation

	Activation			Deactivation			V_{MD}-V_{MA} (mV)	N
	$V_{1/2}$ (mV)	*K*	V_{Median} (mV)	$V_{1/2}$ (mV)	*K*	V_{Median} (mV)		
Control, 22°C	-16.6 ± 2.7	15.3 ± 1.4	-22.5 ± 4.2	-48.4 ± 4.3	21.8 ± 3.6	-42.2 ± 5.5	-10.0 ± 5.0	8
Control, 36°C	-27.7 ± 3.8	20.5 ± 6.3	-31.0 ± 4.2	-21.3 ± 5.3, -52.8 ± 3.6	26.9 ± 4.2, 11.9 ± 1.2	-40.4 ± 4.5	-7.2 ± 5.3	12
scFv2.10, 36°C	-35.5 ± 2.5	16.6 ± 3.0	-36.6 ± 1.2	-66.7 ± 3.1	7.5 ± 1.0	-66.1 ± 4.4*	-22.8 ± 4.3*#	14
scFv2.12, 36°C	-31.8 ± 5.0	17.0 ± 2.3	-31.8 ± 5.0	-68.2 ± 2.8	9.1 ± 1.8	-69.0 ± 10.0*	-34.0 ± 10.0*#	6

*indicates $p < 0.05$ vs Control
#indicates $p < 0.05$ vs "0"

Supplemental Table S2. hERG 1a Ionic Current Activation & Deactivation						
	Activation		Deactivation		$V_{1/2D}$-$V_{1/2A}$ (mV)	N
	$V_{1/2}$ (mV)	*K*	$V_{1/2}$ (mV)	*K*		
Control, 22°C	3.4 ± 4.2*	10.3 ± 0.3*	-55.9 ± 2.8*	12.5 ± 1.2*	-60.2 ± 6.8*#	5
Control, 36°C	-23.3 ± 1.4	7.4 ± 0.4	-46.8 ± 1.4	8.7 ± 0.7	-23.6 ± 1.5#	12
scFv2.10, 36°C	-21.3 ± 1.1	7.0 ± 0.2	-33.8 ± 1.8*	8.7 ± 0.4	-12.5 ± 1.8*#	16
scFv2.12, 36°C	-19.7 ± 1.5	7.5 ± 0.4	-41.4 ± 1.8*	8.5 ± 0.4	-22.3 ± 2.5#	12

*indicates $p < 0.05$ vs Control @ 36°C

Supplemental Table S3. Inducible hERG 1b Cell Line Activation & Deactivation						
	Activation		Deactivation		$V_{1/2D}$-$V_{1/2A}$ (mV)	N
	$V_{1/2}$ (mV)	*K*	$V_{1/2}$ (mV)	*K*		
hERG 1a	-38.7 ± 2.5	8.3 ± 0.5	-60 ± 2.0	11.6 ± 1.4	-20.3 ± 2.2#	9
hERG 1a/1b	-35.9 ± 1.5	8.2 ± 0.6	-49.1 ± 3.4*	9.0 ± 0.5	-13.1 ± 2.6*#	8

*indicates $p < 0.05$ vs hERG 1a

#indicates $p < 0.05$ vs "0"

Supplemental Table S4. Inducible hERG 1b Cell Line Gating Charge Activation & Deactivation								
	Activation			Deactivation			V_{MD}-V_{MA} (mV)	N
	$V_{1/2}$ (mV)	*K*	V_{Median} (mV)	$V_{1/2}$ (mV)	*K*	V_{Median} (mV)		
hERG 1a	-51.4 ± 6.0	22.6 ± 2.6	-42.0 ± 3.6	-35.2 ± 8.3, -82.8 ± 1.7	21.1 ± 4.2, 6.9 ± 2.0	-48.3 ± 6.5	-8.2 ±7.2	6
hERG 1a/1b	-44.0 ± 6.0	17.9 ± 1.0	-41.9 ± 7.7	-72.2 ± 4.5	15.3 ± 1.3	-66.7 ± 5.3*	-36.6 ± 2.6*#	5

*indicates $p < 0.05$ vs hERG 1a

#indicates $p < 0.05$ vs "0"
